# Cutaneous focal mucinosis of the scalp and adenocarcinoma of the lung: association or coincidence?

**DOI:** 10.1590/S1806-37132015000004485

**Published:** 2015

**Authors:** Tiago Mestre, Fernando Assis-Pacheco, Jorge Cardoso

**Affiliations:** 1 Centro Hospitalar de Lisboa Central, Curry Cabral Hospital, Lisbon, Portugal, 1 Intern in Dermatology, Curry Cabral Hospital, Centro Hospitalar de Lisboa Central - CHLC, Central Lisbon Hospital Center - Lisbon, Portugal; 2 Centro Hospitalar de Lisboa Central, Curry Cabral Hospital, Department of Dermatology, Lisbon, Portugal, 2 Senior Consultant, Department of Dermatology, Curry Cabral Hospital, Centro Hospitalar de Lisboa Central - CHLC, Central Lisbon Hospital Center - Lisbon, Portugal; 3 Centro Hospitalar de Lisboa Central, Curry Cabral Hospital, Department of Dermatology, Lisbon, Portugal, 3 Head, Department of Dermatology, Curry Cabral Hospital, Centro Hospitalar de Lisboa Central - CHLC, Central Lisbon Hospital Center - Lisbon, Portugal

## To the Editor:

**Figure 1 -:**
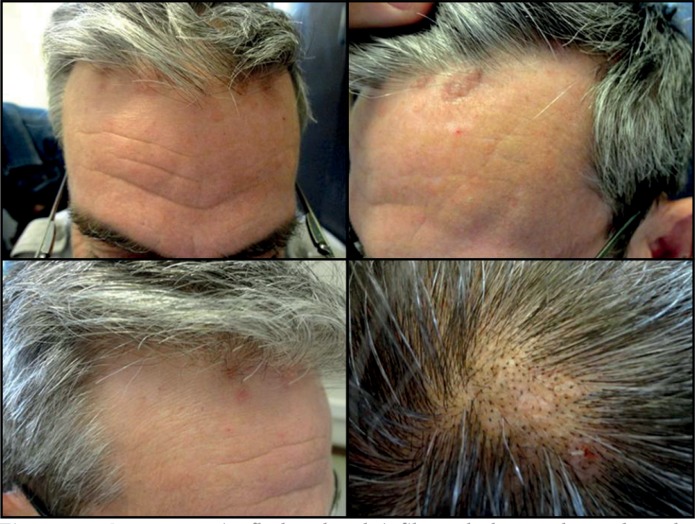
Asymptomatic, flesh-colored, infiltrated plaques, located on the fronto-occipital scalp, which developed over a period of three months.

**Figure 2 -:**
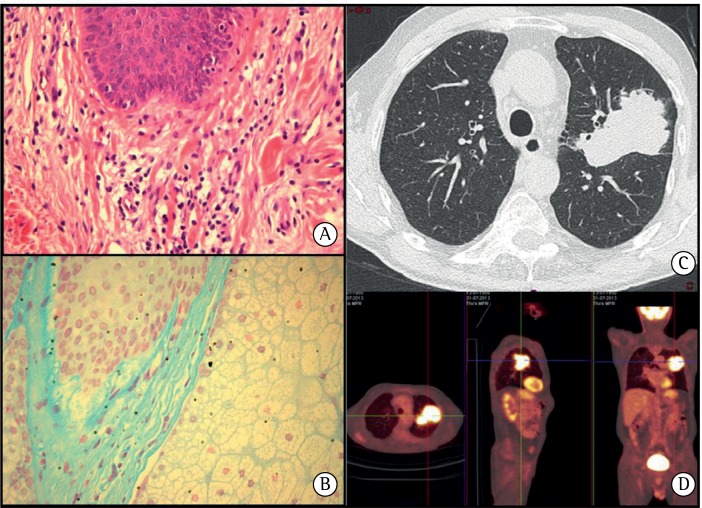
Photomicrographs showing the results of the histopathological staining with H&E (magnification, ×40) and Alcian blue (magnification, ×40), in A and B, respectively. Note the homogeneous deposition of mucin (stained blue) in the dermis, with no alterations of the epidermis or pilosebaceous units, no eosinophils, no epidermotropism, and no granulomas. In C and D, respectively, a CT scan and positron emission tomography scans showing a primary adenocarcinoma in the upper lobe of the left lung.

Here, we present the case of a 62-year-old patient presenting with a three-month history of asymptomatic, flesh-colored, infiltrated plaques on the fronto-occipital scalp (Figure 1). The patient had a history of dyslipidemia and hypertension but reported no changes in medication in the last two years. Serial histopathological examination of the scalp plaques (over a two-year follow-up period) showed moderate dermal lymphocytic infiltrate with homogeneous deposition of mucin in the dermis. There were no alterations of the epidermis or pilosebaceous units, no eosinophils, no epidermotropism, and no granulomas (Figures 2A and 2B). Staining with Alcian blue showed mucin deposits arranged homogeneously in the dermis (Figure 2). The physical examination and the results of extensive laboratory tests (including serum and urine protein immunoelectrophoresis, auto-antibody screening, as well as tests of thyroid, liver, and renal function) were normal. A chest X-ray showed a mass in the upper lobe of the left lung. On the basis of the results of a CT scan and positron emission tomography scans (Figures 2C and 2D), the patient was diagnosed with primary adenocarcinoma of the lung. Examination of a transthoracic biopsy sample resulted in the tumor being classified as stage IIIA (T4N0M0). The patient was referred for cardiothoracic surgery (neo-adjuvant chemotherapy plus surgery with curative intent). At this writing (six months after surgery), there were no signs of recurrence of the adenocarcinoma and no new skin lesions, as well as slight improvement of the existing lesions.

Cutaneous focal mucinosis presents a histological reaction pattern, described in several diseases, in which abnormal focal deposition of mucin is found in the dermis. Cutaneous mucinosis can be classified in several ways: either primary or secondary to an associated disease (including malignancies, connective tissue disorders, and other reactive disorders); by the type of mucin; or by the pattern of distribution of the mucin (focal, follicular, or diffuse). The mucin can also be classified as epithelial or dermal, the two types differing in their composition, as well as in the staining techniques required in order to identify them. Epithelial mucins contain neutral and acid glycosaminoglycans and are hyaluronidase-resistant. They stain positively with periodic acid-Schiff and with Alcian blue at pH 2.5 but fail to stain with toluidine blue. Dermal mucins are periodic acid-Schiff-negative, show metachromatic purple staining with toluidine blue at pH 4.0, stain with Alcian blue at pH 2.5, and are hyaluronidase-sensitive.^(^ 1 ^)^

The etiology of cutaneous focal mucinosis is unknown. It is hypothesized to be a fibroblast disorder in which cytokines or immunoglobulins increase the synthesis of glycosaminoglycan by fibroblasts. The association with malignant disorders, as in our case, might be attributable to cytokine stimulation of fibroblasts and to tumor production of growth factors.^(^ 1 ^)^ We conducted an extensive search of PubMed and found no other reports of cases of adenocarcinoma of the lung accompanied by cutaneous mucinosis.

In cases of focal mucinosis of the scalp, the differential diagnosis should include follicular mucinosis (not always present in alopecia) and mycosis fungoides. In the follicular subtype of mucinosis, the mucin is in the outer root sheath epithelium and sebaceous glands, with lymphocytic infiltrate that is folliculotropic.^(^ 2 ^)^ The deposition of mucin itself is rarely prominent, and it is thought that T cells stimulate the production of mucin by keratinocytes. In the case presented here, we detected mucins only in the dermis and the lymphocytic infiltrate was not folliculotropic.

Treatment for secondary cutaneous focal mucinosis requires treatment of the underlying disease. Although various therapeutic approaches have been tested in cases of primary cutaneous mucinosis, there is no consensus regarding the first-line therapy. Recent studies have indicated that the disease has a chronic course, with recurrent or persistent lesions, in the majority of patients.^(^ 3 ^)^ There is anecdotal evidence of the effectiveness of a number of therapies^(^ 4 ^)^: topical, intralesional, and systemic steroids; topical retinoids; systemic isotretinoin; dapsone; interferon; hydroxychloroquine and cyclophosphamide; methotrexate; psoralen plus ultraviolet A; antihistamines; minocycline; superficial X-ray radiation; photodynamic therapy; and pimecrolimus.

Cutaneous focal mucinosis can have a broad spectrum of clinical presentations. We presented this case in order to illustrate an unusual presentation of cutaneous focal mucinosis of the scalp (without follicular involvement) and to call attention to the importance of ruling out secondary associated malignant disorders, principally in elderly patients, in whom long-term follow-up can be required. To our knowledge, this is the first report of the combination of cutaneous focal mucinosis and adenocarcinoma of the lung.
